# The impact of Omicron pandemic and COVID‐19 vaccination on the pancreatic adenocarcinoma patients

**DOI:** 10.1002/aac2.12056

**Published:** 2022-12-08

**Authors:** Ningzhen Fu, Yu Jiang, Zhiwei Xu, Meng Yang, Chenghong Peng, Xiaxing Deng, Shulin Zhao, Baiyong Shen

**Affiliations:** ^1^ Department of General Surgery Pancreatic Disease Center Ruijin Hospital Shanghai Jiaotong University School of Medicine Shanghai China; ^2^ Research Institute of Pancreatic Disease Shanghai Jiaotong University School of Medicine Shanghai China; ^3^ State Key Laboratory of Oncogenes and Related Genes Shanghai China; ^4^ Institute of Translational Medicine Shanghai Jiaotong University Shanghai China

**Keywords:** lockdown, overall survival (OS), pancreatic adenocarcinoma (PAC), The coronavirus disease‐2019 (COVID‐19)

## Abstract

**Background:**

The coronavirus disease 2019 (COVID‐19) pandemic resulted in enormous medical and economic burden worldwide during the past 3 years. The vaccination was deemed the effective option to prevent the severe symptoms, and especially recommended among cancer patients. Shanghai experienced the first lockdown during the recent Omicron pandemic since 2019. How patients with pancreatic adenocarcinoma (PAC) suffered from the pandemic and how vaccination influenced their oncological outcomes were unexplored yet.

**Method:**

The retrospective study was carried out in a high‐volume referral center including 1157 consecutively enrolled patients with PAC experiencing the COVID‐19 pandemic. The primary outcome was the overall survival (OS).

**Results:**

Limited postoperative patients (9.21%) received the vaccination. The lockdown in Shanghai (April to May, 2022) was not observed impacting the survival prognoses of patients with PAC. Though vaccination was not significantly associated with OS itself (adjusted hazard ratio (aHR): 2.032 [0.940–4.391], *p =* 0.071), it was discovered to synergistically improve the chemotherapy effect in the multivariate analyses (interaction *p =* 0.023).

**Conclusion:**

The vaccination itself did not influence the survival prognoses of patients with PAC. A potential positive interaction was observed between chemotherapy and vaccination despite the limited follow‐up time. The postoperative patients should consider the vaccination more. The patients with PAC did not suffer worse prognostic outcomes from the strict sanitary policy during the wave of COVID‐19 pandemic in Shanghai.

## Graphical abstract

A potential positive interaction was observed between chemotherapy and vaccination. The chemotherapy performed well among vaccinated patients but could not improve the OS among unvaccinated patients. Sole vaccination without chemotherapy manifested a risk factor for patients’ OS, suggesting that the vaccinated patients should preferably receive the chemotherapy.

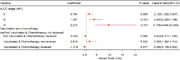

## 1 BACKGROUND

Coronavirus disease 2019 (COVID‐19) pandemic has lasted for over 3 years, resulting in over 528 million infections and 6 million deaths worldwide.^1^ COVID‐19, caused by the severe acute respiratory syndrome coronavirus 2 (SARS‐CoV‐2) virus, is a highly contagious disease through human‐to‐human transmission.^2^ The transmission mechanism of SARS‐CoV‐2 is reported via respiratory droplets and direct contact.^3^ The reproductive ratio for SARS‐CoV‐2 was ever reported between 1.4 and 2.5, which could cause an exponential infectious increase.^2^ Notably, considering certain patients who could be infectious before symptoms appeared (presymptomatic), and the asymptomatic patients (30%–60% of total^4^), the risk of human‐to‐human transmission could further increase.^5^ The elderly and the preexisting medical condition were discovered risk factors of the severe viral pneumonia and the progression to severe acute respiratory syndrome (SARS) or eventual death.^3^ The Omicron variant, the latest one of SARS‐CoV‐2, was reported less virulent but more contagious.^6^ The Omicron variant spread across the world and became dominant within half a year.^7^

Several therapies in the general population such as dexamethasone, Janus kinase 1/2 inhibitor, and monoclonal antibodies have been explored. Nevertheless, the benefits, costs, and adverse effects were not satisfactory enough.^8^, ^9^, ^10^, ^11^ In view of the critical pandemic situation, vaccination was deemed an effective approach to prevent the infection and reduce the mortality.^12^ The vaccine types mainly included mRNA (e.g., BNT162b2, mRNA1273), viral vector (e.g., ChAdOx1), and inactivated virus (e.g., CoronaVac, COVILO). Because cancer itself manifested as an independent risk factor for poor COVID‐19 prognosis,^13^ international organizations such as the National Comprehensive Cancer Network and the Asian Oncology Society and the European Society for Medical Oncology have urged the prioritization of COVID‐19 vaccination among cancer patients.^14^, ^15^ However, due to the limited physical status or the worry about safety of the vaccine, many cancer patients refuse the vaccination, which led to the insufficient epidemic prevention.^16^ It is a critical challenge for public health and policy execution. Pancreatic adenocarcinoma (PAC), one of the most lethal malignancies,^17^ is closely associated with abnormal immune function.^18^ It is unclear whether the impact of vaccination on the immune system influenced the oncological status of patients with PAC or not. The rate of vaccination and the association between the vaccination and oncological outcome have not been explored among patients with PAC.

Recently, a wave of COVID‐19 Omicron infection broke out in Shanghai. According to the Shanghai Municipal Health Commission, 593,336 cases have been identified, including 503 people who died with or of COVID‐19 by May 4, 2022.^19^ The lockdown lasting over 2 months was implemented in the 25,000,000 population city. The patients with PAC faced tremendous challenges including the interruption of the chemotherapy, the reduced postoperative care, the hindered outpatient service, and the follow‐up works. How the patients with PAC suffered from this pandemic wave needs to be restudied. In this study, we tried to answer the questions above with a large consecutive cohort from a high‐volume referral PAC center in Shanghai.

## 2 METHOD

### 2.1 Data collection

Target population was alive or died during January 2019 to May 2022 (experiencing the COVID‐19 pandemics) with regular follow‐ups, who were diagnosed as PAC from January 2010 to May 2022. All eligible patients were consecutively enrolled from the Pancreatic Disease Center, Ruijin Hospital Affiliated to Shanghai Jiao Tong University School of Medicine. The inclusive criteria were as follows: (1) pathologically diagnosed as PAC, (2) aged between 18 and 85 years old and (3) receiving curative pancreatectomy or palliative pancreatic surgery. The exclusion criteria were as follows: (1) incomplete oncological data (including pathological American Joint Committee of Cancer (AJCC) stage for curative pancreatectomy patients and radiological AJCC stage for palliative pancreatic surgery patients), (2) without regular follow‐up, and (3) heterogenous carcinoma.

The study protocol was approved by the Institutional Review Board of the authors’ affiliated hospital. The local ethics committee waived the need for informed consent because the study was observational and retrospective. The study was conducted according to the Strengthening the Reporting of Observational Studies in Epidemiology (STROBE) guidelines and in accordance with the latest version of the Declaration of Helsinki.

### 2.2 Statistical analysis

The primary outcome was the overall survival (OS). Normally distributed continuous variables are presented as mean ± SD and analyzed using Student's *t*‐test. Non‐normally distributed continuous variables were presented as median (Q1–Q3) and analyzed using the Mann–Whitney *U* test. The Kolmogorov–Smirnov test was used for the normality tests of continuous variables. Categorical variables are presented as percentages and analyzed using Pearson's test. The Wilcoxon sum rank test was used for pair‐wise comparisons between non‐normally distributed factors. OS was assessed using Cox proportional hazards models in multivariate analyses adjusted for clinically relevant factors identified in the univariate analyses. The hazard ratio (HR) and adjusted hazard ratio was displayed with a 95% confidence interval (CI). The Cox–Mantel test was used for significance comparison. The multiplicative interaction was displayed by the product term in multivariate models. The additive interaction was displayed by the relative excess risk due to interaction (RERI) and attributable proportion due to interaction (AP). The association between categorical data was assessed using the *χ* ^2^ test, while the association between continuous data was assessed using the Spearman rank test, and the association between categorical data and continuous data was assessed using the Kruskal–Wallis test.

Statistical analysis was performed using R statistical suite (R Core Development Team). *p* < 0.050 was regarded as statistically significant.

## 3 RESULTS

### 3.1 Patients and characteristics

**FIGURE 1:**
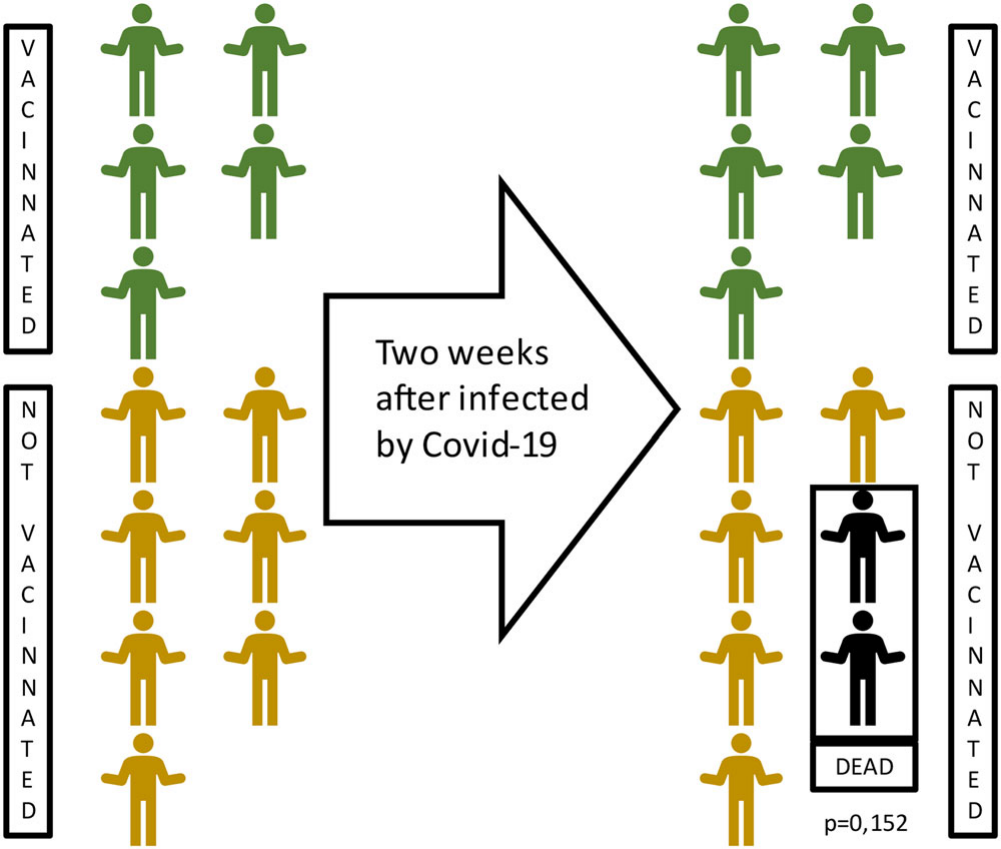
Sample graph of coronavirus disease 2019 (COVID‐19) positive pancreatic adenocarcinoma (PAC) patients

A total of 1157 patients were enrolled in our study. The median follow‐up period was 15.13 months in total. The baseline data are displayed in Table 1. Except for AJCC stage, no significant differences were observed between the vaccinated (vac) and nonvaccinated (novac) groups. The patients in the vac group were observed with more advanced disease. The adjuvant chemotherapy ratio was 66.21% in total. Twelve patients were infected with COVID‐19. Notably, the only two deaths among the 12 deaths both belonged to the novac group (Figure 1).

**TABLE 1:** Demographic and baseline characteristics

		No vac = 821		Vac = 336		*p*
Age^a^		62.97	9.55	61.95	9.52	0.99
Gender^b^	Female	350	42.63%	129	38.39%	
	Male	471	57.37%	207	61.61%	0.207
BMI^a^		22.92	3.02	23.10	2.94	0.362
WBC^a^		6.02	2.17	6.08	1.86	0.643
HB^a^		127.72	16.79	126.38	20.26	0.285
PLT^a^		206.34	72.97	200.03	66.64	0.156
TB^c^		14.55	[10.60; 46.38]	14.60	[10.00; 44.82]	0.800
Alb^a^		40.14	4.67	39.82	5.06	0.317
Amy^c^		61.50	[43.00; 95.00]	62.00	[44.00; 101.00]	0.509
CA199^c^		120.1	[34.05; 406.55]	133.05	[34.98; 414.85]	0.550
CEA^c^		2.91	[1.73; 4.85]	2.75	[1.60; 4.56]	0.333
AJCC^b^	I	337	41.05%	105	31.25%	
	II	290	35.32%	129	38.39%	
	III	160	19.49%	85	25.30%	
	IV	34	4.14%	17	5.06%	0.012
Chemotherapy^b^	Yes	534	65.04%	232	69.05%	
	No	132	16.08%	79	23.51%	
	Unk	155	18.88%	25	7.44%	0.059
COVID‐19 positive^b^	7	0.85%	5	1.49%	0.346	

Abbreviations: AJCC, American Joint Committee of Cancer stage; Alb, albumin; Amy, amylase; BMI, body mass index; CEA, carcinoma embryonic antigen; COVID‐19, coronavirus disease 2019; HB, hemoglobin; no vac, the nonvaccinated group; PLT, platelet; TB, total bilirubin; Unk, unknown; Vac, the vaccinated group; WBC, white blood cell.

^a^ Mean (standard deviation).

^b^ Number of patients, *n* (percentage).

^c^ Median (quantile).

### 3.2 Vaccination and death during COVID‐19 pandemic

**FIGURE 2:**
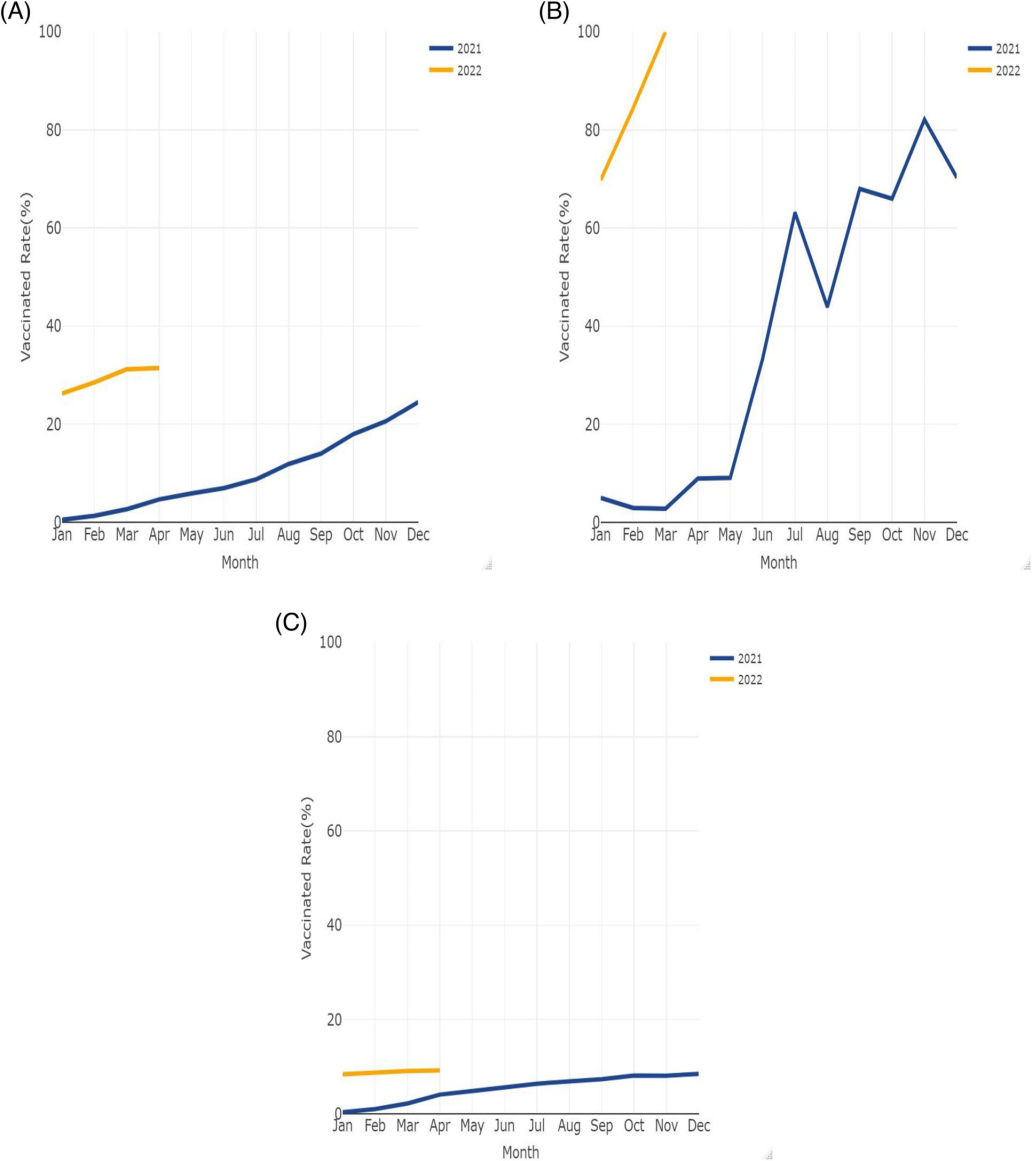
(A) Vaccination rate in the alive patients (vaccinated before or after pancreatic adenocarcinoma (PAC) diagnosis). (B) Vaccination rate at the time of diagnosis (vaccinated before PAC diagnosis). (C) Vaccination rate of the alive patients who were not vaccinated when PAC diagnosis (vaccinated after PAC diagnosis).

**FIGURE 3:**
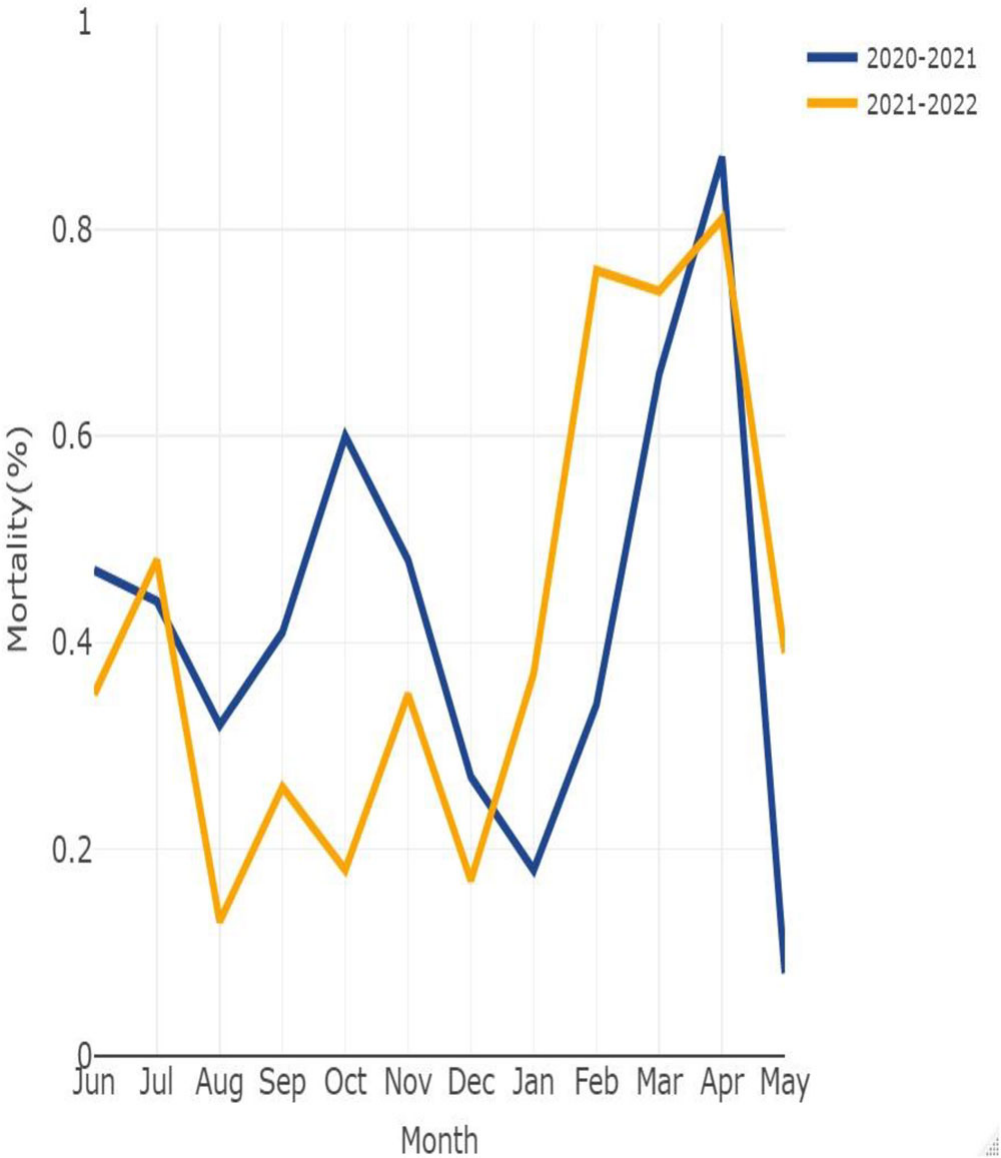
Monthly mortality of all the alive patients pooled

During the COVID‐19 pandemic, 336 patients were vaccinated. Among whom, 263 patients were vaccinated before diagnosis and 73 after diagnosis. The number of patients who received one vaccination, one vaccinations, and booster vaccination were 30, 224, and 82, respectively. The vaccination rate is displayed in Figure 2. The vaccination rate increased steadily for all of the alive patients in our cohort from the introduction of vaccination and just reached 31.43% recently by April 2022 (Figure 2A). The vaccination rate before the diagnosis of PAC increased quickly in the last year and reached 100% recently just before the wave (Figure 2B). The vaccination rate of the alive postoperative patients, who were not vaccinated when diagnosed with PAC, was only 9.21% by April 2022 (Figure 2C). The monthly deaths presented in Figure 3 showed similar rates in the last 2 years.

### 3.3 Adjuvant chemotherapy improved the OS among vaccinated patients

**FIGURE 4:**
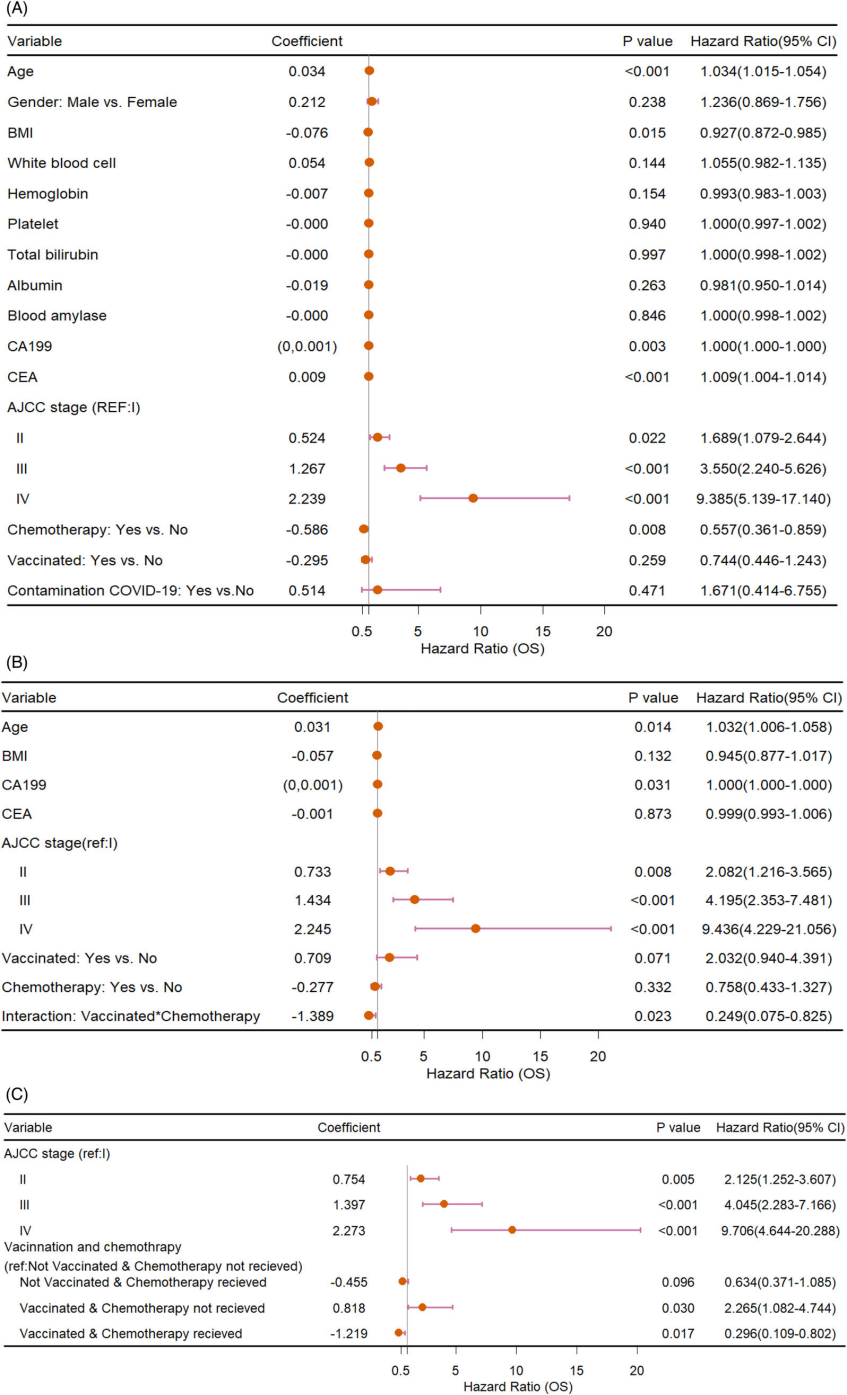
(A) Forest plot of the univariate COX analyses. (B) Forest plot of the multivariate COX model. (C) Multivariate model included categorical vaccination and chemotherapy situation. Abbreviations: BMI, body mass index; OS, overall survival; CI, confidence interval

Age, body mass index, CA199, carcinoma embryonic antigen (CEA), AJCC stage, and adjuvant chemotherapy administration were discovered to be significantly associated with OS in univariate analyses (Figure 4A). In the multivariate model, the product term of adjuvant chemotherapy and vaccination were significant, indicating the existence of multiplicative interaction (Figure 4B). The additive interaction was then verified (RERI [95% CI]: 1.38 [0.28–2.47]; AP [95% CI]: 0.83 [0.34–1.31]). How the vaccination interacted with adjuvant chemotherapy was explored (Figure 4C).

## 4 DISCUSSION

Despite the international associations recommending the vaccination to cancer patients,^14^, ^15^ the impact of vaccines on cancer patients was still misty. In this study, we sought to elaborate on how vaccination influenced the health status and survival prognoses of patients with PAC.

We screened 1157 patients who experienced the COVID‐19 pandemic. The vaccination was introduced as emergency use to the public at the beginning of 2021 with insufficient phase III and IV clinical trial results. The vaccination rate kept increasing since then. Comparing with the quickly increasing vaccination rate of newly diagnosed patients, the rate of post‐treatment patients was still unfavorable. Among the total cohort, 12 patients were diagnosed as COVID‐19 positive. Two patients died among them and both belonged to the novac group. A previous study also reported that COVID‐19 infection was significantly associated with the increased perioperative morbidity and mortality.^20^

A strict lockdown was imposed in Shanghai from the beginning of April to the beginning of June 2022. Despite the strict sanitary policy, which impeded the subsequent visits and treatments of patients with PAC treated in our center, the chemotherapy was introduced to them in the local hospitals with sufficient communications between our center and the primary care hospitals. These efforts minimized the adverse impact of lockdown on patients with PAC. In Figure 3, the trend of monthly death during the pandemic wave was similar to that in 2021, indicating that the impact of lockdown on the survival of patients with PAC was limited in the short term. However, continuous follow‐ups should be processed to further observe in a long term. Some studies proposed that the COVID‐19 could impair the prognoses of pancreatic cancer patients,^21^, ^22^ which probably resulted from the impaired cancer screening, diagnosis, and care.^23^

The vaccination was not discovered associated with the OS of PAC patients. However, with further interaction analyses, vaccination was observed to have interacted with the adjuvant chemotherapy administration. Both additive and multiplicative interactions were observed. Relying on further subgrouping, multivariate analysis revealed that chemotherapy performed well among vaccinated patients but could not improve the OS among unvaccinated patients. Considering the sample size and limited follow‐up period (not even reaching the median OS), the impact of chemotherapy on unvaccinated patient subgroup could be insignificant. Sole vaccination without chemotherapy manifested a risk factor for patients’ OS, suggesting that the vaccinated patients should preferably receive chemotherapy. Notably, the limited number for vaccination (+) chemotherapy (‐) patients might influence the reliability of the result. The mechanism beneath the positive interaction between chemotherapy and vaccination could be complicated. First, the vaccinated patients were probably more compliant. Second, the vaccination could help activate the immune system and further synergistically improve the effect of chemotherapy.^24^ Third, the unvaccinated patients worried about the infection of COVID‐19 and were thereby less likely to visit the hospital and receive the medical treatments.^23^

Some limitations of the study should be emphasized. First, it is a retrospective study with a certain uncorrected bias. Second, the chemotherapy regimen was not discussed to avoid excessive subgrouping in the limited total cohort. Third, the mechanism beneath the positive interaction were not explored furthermore.

In this study, we found that the vaccination itself did not influence the survival prognoses of patients with PAC. A potential positive interaction was observed between chemotherapy and vaccination despite the limit of the follow‐up time. The postoperative patients should consider the vaccination more. The patients with PAC did not suffer worse oncological outcome from the strict sanitary policy during the wave of the COVID‐19 pandemic in Shanghai.

## AUTHOR CONTRIBUTIONS

*Study concepts*: Yu Jiang, Shulin Zhao, and Baiyong Shen. *Study design*: Ningzhen Fu, Shulin Zhao, and Xiaxing Deng. *Data acquisition*: Yu Jiang and Meng Yang. *Quality control of data and algorithms*: Shulin Zhao, Zhiwei Xu, and Xiaxing Deng. *Data analysis and interpretation*: Ningzhen Fu and Shulin Zhao. *Statistical analysis*: Shulin Zhao and Chenghong Peng. *Manuscript preparation*: Ningzhen Fu and Xiaxing Deng. *Manuscript editing*: Ningzhen Fu, Shulin Zhao, and Baiyong Shen. *Manuscript review*: Xiaxing Deng, Chenghong Peng, and Baiyong Shen.

## CONFLICT OF INTEREST

Dr. Shen is Editor‐in‐Chief of the journal and co‐author of this article. They were excluded from the peer‐review process and all editorial decisions related to the acceptance and publication of this article. Peer‐review was handled independently by the other EiC to minimize bias. Other authors report no conflict of interest.

## FUNDING INFORMATION

This study did not receive any funding.

## ETHICS STATEMENT

This study was exempt from institutional review board approval due to the nature of the study. Since all data were deidentified, patient consent was waived.

## Data Availability

The data from where our results derived were from Pancreatic Disease Center, Shanghai Jiao Tong University School of Medicine Affiliated Ruijin Hospital. The original data were not publicly available and could only be shared with the permission of the ethics committee of Ruijin Hospital.
